# Blood DNA methylation signatures of lifestyle exposures: tobacco and alcohol consumption

**DOI:** 10.1186/s13148-022-01376-7

**Published:** 2022-11-28

**Authors:** Jonviea D. Chamberlain, Sébastien Nusslé, Laurence Chapatte, Cassandre Kinnaer, Dusan Petrovic, Sylvain Pradervand, Murielle Bochud, Sarah E. Harris, Janie Corley, Simon R. Cox, Semira Gonseth Nusslé

**Affiliations:** 1 Department of Epidemiology and Health Systems (DESS), University Center for General Medicine and Public Health (Unisanté), Route de la Corniche 10, 1010 Lausanne, Switzerland; 2 Genknowme, Epalinges, Switzerland; 3 Vital-IT Group, Swiss Institute of Bioinformatics, Lausanne, Switzerland; 4 Genomic Technologies Facility, University of Lausanne, Lausanne, Switzerland; 5 Lothian Birth Cohorts, Department of Psychology, University of Edinburgh, Edinburgh, UK

**Keywords:** Epigenetic signature, Tobacco consumption, Alcohol consumption, Lifestyle exposures, Epigenetic epidemiology

## Abstract

**Background:**

Smoking and alcohol consumption may compromise health by way of epigenetic modifications. Epigenetic signatures of alcohol and tobacco consumption could provide insights into the reversibility of phenotypic changes incurred with differing levels of lifestyle exposures. This study describes and validates two novel epigenetic signatures of tobacco (EpiTob) and alcohol (EpiAlc) consumption and investigates their association with disease outcomes.

**Methods:**

The epigenetic signatures, EpiTob and EpiAlc, were developed using data from the Swiss Kidney Project on Genes in Hypertension (SKIPOGH) (*N* = 689). Epigenetic and phenotypic data available from the 1921 (*N* = 550) and 1936 (*N* = 1091) Lothian Birth Cohort (LBC) studies, and two publicly available datasets on GEO Accession (GSE50660, *N* = 464; and GSE110043, *N* = 94) were used to validate the signatures. A multivariable logistic regression model, adjusting for age and sex, was used to assess the association between self-reported tobacco or alcohol consumption and the respective epigenetic signature, as well as to estimate the association between CVD and epigenetic signatures. A Cox proportional hazard model was used to estimate the risk of mortality in association with the EpiTob and EpiAlc signatures.

**Results:**

The EpiTob signature was positively associated with self-reported tobacco consumption for current or never smokers with explained variance ranging from 0.49 (LBC1921) to 0.72 (LBC1936) (pseudo-*R*^2^). In the SKIPOGH, LBC1921 and LBC1936 cohorts, the epigenetic signature for alcohol consumption explained limited variance in association with self-reported alcohol status [i.e., non-drinker, moderate drinker, and heavy drinker] (pseudo-*R*^2^ = 0.05, 0.03 and 0.03, respectively), although this improved considerably when measuring self-reported alcohol consumption with standardized units consumed per week (SKIPOGH *R*^2^ = 0.21; LBC1921 *R*^2^ = 0.31; LBC1936 *R*^2^ = 0.41). Both signatures were associated with history of CVD in SKIPOGH and LBC1936, but not in LBC1921. The EpiTob signature was associated with increased risk of all-cause and lung-cancer specific mortality in the 1936 and 1921 LBC cohorts.

**Conclusions:**

This study found the EpiTob and EpiAlc signatures to be well-correlated with self-reported exposure status and associated with long-term health outcomes. Epigenetic signatures of lifestyle exposures may reduce measurement issues and biases and could aid in risk stratification for informing early-stage targeted interventions.

**Supplementary Information:**

The online version contains supplementary material available at 10.1186/s13148-022-01376-7.

## Introduction

Lifestyle exposures, such as smoking and alcohol consumption, are known to play a major role in health and, as such, are often a focus in epidemiological studies and targeted health interventions. Unfortunately, given that such lifestyle exposures are usually collected by self-report questionnaires they can be subject to information bias [1]. Biomarkers can aid in differentiating exposure status and thereby help mitigate potential biases, for example, urinary cotinine to assess exposure to smoking [2]. However, individual biomarkers often require separate blood or urine assays that, in addition to clinical sample collection, can contribute to financial burden at the study-level, but also patient-level burden. Moreover, while the status of the exposure (e.g., smoker versus non-smoker) matters, the extent of damage incurred at a molecular level may be what leads to disease and may vary across people depending on various genetic and/or environmental factors.

Smoking and alcohol consumption may compromise health by way of epigenetic modifications that alter gene expression and cellular function and can in turn cause changes to phenotype expression. One form of epigenetic modifications—DNA methylation—can be measured using genome-wide arrays. Depending on the location of the methylated DNA, these modifications can alter gene expression, including gene silencing. Importantly, epigenetic changes induced by DNA methylation can be reversible. Quantifying DNA methylation in white blood cells associated with lifestyle exposures is thus useful not only for risk-stratification, but also in relation to personalized medicine and developing dynamic, individualized prevention strategies.

Polyepigenetic risk scores—or epigenetic signatures—of alcohol and tobacco consumption could provide insights into the reversibility of phenotypic changes incurred with differing levels and duration of lifestyle exposures. Moreover, epigenetic signatures of tobacco and alcohol consumptions could serve as biological proxies for self-reported exposure status to avoid reporting bias and help enrich study data given the multitude of uses for epigenetic information once collected that goes beyond any single signature estimation. Using data from the SKIPOGH cohort—a population-based cohort study with extensive data on lifestyle exposures, genetic and epigenetic information—the objective of this study is to create two novel epigenetic signatures of tobacco (EpiTob) and alcohol (EpiAlc) consumption using evidence from the literature and SKIPOGH data, to validate them using independent databases and to investigate their association with CVD and all-cause and cause-specific mortality.

## Methods

### Study population

This study used data collected from individuals included in the Swiss Kidney Project on Genes in Hypertension (SKIPOGH) study [3]. Briefly, SKIPOGH is a multi-center, family-based cohort study of participants recruited between 2009 and 2013; a follow-up (wave 2 [W2]) occurred between 2012 and 2016 [3]. At both waves, data were collected on sociodemographic information, health status (including medical history), lifestyle and sleeping habits, physical activity, as well as clinical and anthropometric exam results. Inclusion criteria consisted of (1) written informed consent, (2) European descent, (3) 18 years of age or older, as well as (4) the additional inclusion of at least one first-degree family member [3]. Data on DNA methylation were only collected for a sub-sample of participants at W2. The analytical sample of the present study is therefore restricted to information collected at W2.

### Dependent variables of interest

Information on alcohol and tobacco consumption status collected during the follow-up wave of SKIPOGH was used in the present study. Alcohol consumption was defined based on the Swiss Federal Public health guidelines on prevention of alcohol abuse (www.addictionsuisse.ch) using the self-reported average number of recorded alcohol units (1-unit ≈ 10 g of pure alcohol) consumed per week. Self-reported alcohol consumption status categorized into heavy (> 21 units per week for men, > 14 units for women), moderate (1–21 units for men, 1–14 units for women) and non-drinkers (less than 1 unit of alcohol consumed per week). Self-reported smoking status (including all forms of tobacco consumption) was categorized into three categories: current smoker, ex-smoker, and non-smoker. Cardiovascular-related disease (CVD) status (yes/no) was defined as ever having had a cardiovascular event, including coronary heart disease, stroke, or any other cardiovascular-related event (e.g., coronary disease, angina, infarct).

### Covariates

Information collected during the follow-up wave of SKIPOGH on age, sex, body mass index (BMI), educational attainment, alcohol and tobacco consumption status was used in the present study.

### Pre-processing of DNA methylation data

DNA was extracted from white blood cells of SKIPOGH participants using a bead-based KingFisher Duo robot extraction system (ThermoFisher, Waltham, Massachusetts), with 1.2 µg of DNA then treated with bisulfite using the EZ DNA Methylation© Kit (Zymo Research). Alternative incubation conditions (described in the EZ DNA Methylation™ Kit bisulfite conversion protocol, point 6 of the appendix) were used when performing the polymerase chain reaction (PCR) using the Illumina Infinium© Methylation Assay, and the final elution was carried out using 8 µl of M-Elution Buffer. DNA methylation levels were then assessed using genome-wide DNA methylation micro-array platforms Illumina HumanBeadChip 450 K (HM450K) (*N* = 250) and EPIC 850 K (EPIC) (*N* = 480). Missing CpG data were imputed using the nearest averaging multiple imputation method, beta coefficients were then logit transformed [4, 5]. The CPACOR pipeline was used to normalize data and identify quality control issues [6]. Samples with issues identified in a quality control step, e.g., a call rate of less than 95% were excluded from all analyses (*p* value < 10^–16^) (HM450k: *N* = 9; EPIC: *N* = 15). CpGs identified as being present across both the EPIC and HM450k arrays (*N* = 452,453) were subsequently used for all analyses and calculations of epigenetic signatures.

### Epigenetic signatures for tobacco and alcohol consumption

Relevant CpG sites were identified in a scoping literature review of epigenome-wide association studies (EWAS)—2019 or prior—published in the EWAS atlas [7], including all CpGs associated with the traits “smoking” or “alcohol consumption”; only those CpGs with Bonferroni-level significance were retained for inclusion in models [8, 9]. Potential genetic confounding factors (i.e., single nucleotide polymorphisms [SNPs] in close proximity to CpG sites) were identified by testing whether SNPs were methyl-quantitative trait loci (mQTL) in SKIPOGH data according to associated GWAS [10]. As a result, 241 CpG loci and 22 associated methyl-quantitative trait loci SNPs were identified (according to methodology as described by Gonseth et al. [11]) for tobacco consumption, and 57 CpG loci and 2 SNPs for alcohol consumption. Models were then generated to include random combinations of seven CpG sites at a time, including one CpG and its associated SNP; the CpG sites and SNPs were included as exposure variables and the trait (i.e., smoking or alcohol consumption status) as the outcome. CpGs were selected from an initial list of 325 CpGs for tobacco consumption, and 63 for alcohol consumption. Models were restricted to a binary outcome of smoking status (smoker versus never smoker) and drinking status (non-drinker versus drinker). The model that maximized the goodness-of-fit for each respective trait of interest—according to the Bayesian Information Criterion (BIC) —was chosen to create the final epigenetic signature, with the hypothesis being that the model with the best fit will have the greatest biological relevance. Three separate CpG sites were identified for the epigenetic signature of alcohol consumption (EpiAlc), namely: cg06690548, cg03497652, and cg00716257. For the epigenetic signature of tobacco consumption (EpiTob), five different CpG sites were identified: cg05575921, cg26703534, cg23480021, cg08118908, cg00336149. No SNP was identified for inclusion in either of the final predictive models. The number of identified CpGs is due to the initial restriction of a maximum of seven included CpGs, whereby the resulting number of CpGs being fewer than seven is a consequence of the regression approach used, and the inclusion of only those CpGs identified as being significantly associated with the outcome of interest.

### Validation cohorts

Two external validation datasets and data from two independent cohort studies were used to validate the epigenetic signatures for tobacco and alcohol consumption developed with SKIPOGH data. Relevant replication datasets were identified in the Gene Expression Omnibus data repository: the “GSE50660” (tobacco consumption) (Illumina Infinium HumanMethylation450 BeadChip) and the “GSE110043” (alcohol consumption) (Illumina InfiniumEPIC Human Methylation Beadchip) datasets. Included in the GSE50660 dataset is information on DNA methylation from 464 people of European descent who were identified based on participation in a coronary artery disease study across three different centers (Paris, France; Leicester and Cambridge, England) [12]. Meanwhile, the GSE110043 cohort includes 47 cases of individuals admitted consecutively for alcohol detoxification to one of three substance use treatment organizations (Iowa, U.S.A.) and 47 abstinent controls, or individuals who abstained from drinking during the year preceding study inclusion [8]. Whole blood DNA samples were acquired upon study inclusion, which occurred between 1 and 7 days post-admittance for cases [8].

Data from the Lothian Birth Cohorts of 1921 (LBC1921) and 1936 (LBC1936) [9] were used to (a) validate the epigenetic signatures as a biomarker of exposure, and (b) assess the validity and replicability of associations observed in the SKIPOGH cohort study between individual epigenetic signatures and disease outcome. Most of the people included in both LBC cohorts are of European descent. The present study uses DNA methylation in white blood cells, clinical and questionnaire variables of self-reported exposures collected during the first follow-up for each cohort (Wave 1) [10]. Normalization of the DNA methylation data was performed according to internal controls and background subtraction [15]. In both the 1921 and 1936 cohorts, units of alcohol consumption and tobacco consumption were similarly categorized as described above. CVD was defined in the Lothian birth cohorts as “history of cardiovascular disease” (yes/no) and based on the question “Have you ever had a heart attack, angina, heart valve problem, abnormal heart rhythm, or any other heart problem?” Information on vital status up until February 2021 was acquired via linkage to the National Health Service Central Register in Scotland. Cause-specific mortality was classified based on the primary ICD-10 (International Classification of Disease, revision 10) code recorded. ICD-10 codes for cardiovascular disease- and lung cancer-related mortality can be found in the additional files (*Additional file **1*). Due to differences in data collection, population characteristics and variable availability, data for the two LBC cohorts were analyzed separately.

### Statistical analyses

Descriptive statistics of SKIPOGH participant characteristics are provided stratified according to the availability of DNA methylation data. Characteristics of the analytical sample (i.e., excluding those without DNA methylation data or with missing data related to the smoking or alcohol exposure) are provided stratified according to cohort. To assess the relationship between self-reported tobacco or alcohol consumption and the respective epigenetic signature (EpiTob and EpiAlc), a multivariable logistic regression model was used, including self-reported exposure as the binary dependent variable and the epigenetic signature as the independent variable of interest; models were adjusted for age and sex. McFadden’s pseudo-*R*^2^ is reported to measure the explained variation [11].

Logistic regression models were used to investigate the association between EpiTob and EpiAlc and CVD, individually. Model diagnostics included (a) ensuring the linear effect of the continuous exposure on the outcome, (b) checking for influential values using Cook’s distance, and (c) multicollinearity. When using SKIPOGH data, mixed effects models that included center and family ID as fixed effects, separately, were used in a sensitivity analysis to ensure that center and potential family-level exposures did not change results. In addition, inverse probability weights (IPWs) based on availability of epigenetic data for SKIPOGH participants were used in a sensitivity analysis to ensure that potential selection bias did not affect conclusions.

By virtue of the extended follow-up and comprehensive vital status assessment in the LBC cohorts, Cox proportional hazard models were used to assess the risk of mortality in association with lifestyle exposures. Hazard ratios (HRs) and 95% confidence intervals (CIs) are reported. Potential violations of the proportional hazard assumption were assessed via the visual inspection of Kaplan–Meier curves and testing of scaled Schoenfeld residuals. In case of violations, potential nonlinearity of continuous variables was assessed using penalized splines [*pspline* in R] and three degrees of freedom. Competing risk analyses were carried out using the *mstate* package using a transition matrix composed of two absorbing states: (1) the cause-specific outcome of interest and (2) all other causes of death. Subhazard ratios (sHRs) are reported with 95% confidence intervals [17]. The proportional hazard assumption using the same methodology as described above. Regression results for the EpiTob and EpiAlc signatures are presented as standardized values, such that a one-unit increase in the signature corresponds to an increase in one standard deviation.

Analyses were carried out using R Studio (R version 4.0.2) [18].

## Results

### Descriptive statistics

Epigenetic signatures were estimated for 701 SKIPOGH participants (of 1034; 67.8%), of which 689 had available data on alcohol or tobacco consumption. In comparison to excluded participants, participants were older and had a higher BMI (*p* < 0.05), with a greater proportion of smokers or past smokers (*Additional file **1*). No differences were observed according to alcohol consumption or sex. Compared to the validation cohorts, SKIPOGH participants were younger, had a lower BMI, included a greater proportion of females, current or past smokers, and had an overall lower average number of alcohol units consumed per week (Table 1).

**Table 1:** Study Characteristics of analytical sample, stratified by cohort

	LBC1921 (*N* = 550)	LBC1936 (*N* = 1091)	SKIPOGH (*N* = 689)
*Age (years)*			
Mean (SD)	79.3 (0.6)	69.73 (0.8)	52.5 (15.5)
Q1, Q3	78.9, 79.7	69.1, 70.4	41.2, 65.3
Missing	0	0	0
*Sex*			
Male	238 (41.8%)	548 (50.2%)	330 (48.1%)
Female	331 (58.2%)	543 (49.8%)	356 (51.9%)
*Body mass index (kg/m^2)*			
Mean (SD)	26.2 (4.2)	27. 8 (4.4)	25.64 (4.7)
Q1, Q3	23.4, 28.5	24.9, 30.3	22.3, 28.2
Missing	6	2	1
*Alcohol unit(s) consumed per week*			
Mean (SD)	8.7 (10.1)	10.5 (14.2)	6.4 (9.0)
Q1, Q3	2.0, 13.0	0.5, 15.0	0.0, 9.1
Missing	0	0	0
*Alcohol status*			
Non-drinker	137 (24.9%)	211 (19.3%)	204 (29.9%)
Moderate drinker	333 (60.5%)	686 (62.9%)	413 (60.6%)
Heavy drinker	80 (14.5%)	194 (17.8%)	65 (9.5%)
*Smoking status*			
Non-smoker	238 (43.4%)	501 (45.9%)	288 (42.0%)
Past smoker	271 (49.4%)	465 (42.6%)	224 (32.7%)
Current Smoker	40 (7.3%)	125 (11.5%)	174 (25.4%)
*ES: tobacco*			
Mean (SD)	− 0.1 (2.3)	0.8 (2.8)	− 1.5 (2.6)
Q1, Q3	− 1.4, 1.1	− 1.0, 2.0	− 3.0, − 0.6
Missing	115	196	0
*ES: alcohol*			
Mean (SD)	7.2 (6.6)	7.6 (7.4)	8.0 (4.8)
Q1, Q3	3.4, 9.7	2.8, 10.8	5.1, 9.4
Missing	115	196	0
*History of CVD*			
No CVD	382 (81.3%)	823 (75.4%)	560 (79.3%)
CVD history	88 (18.7%)	268 (24.6%)	146 (20.7%)

### Goodness-of-fit in SKIPOGH

**Fig. 1:**
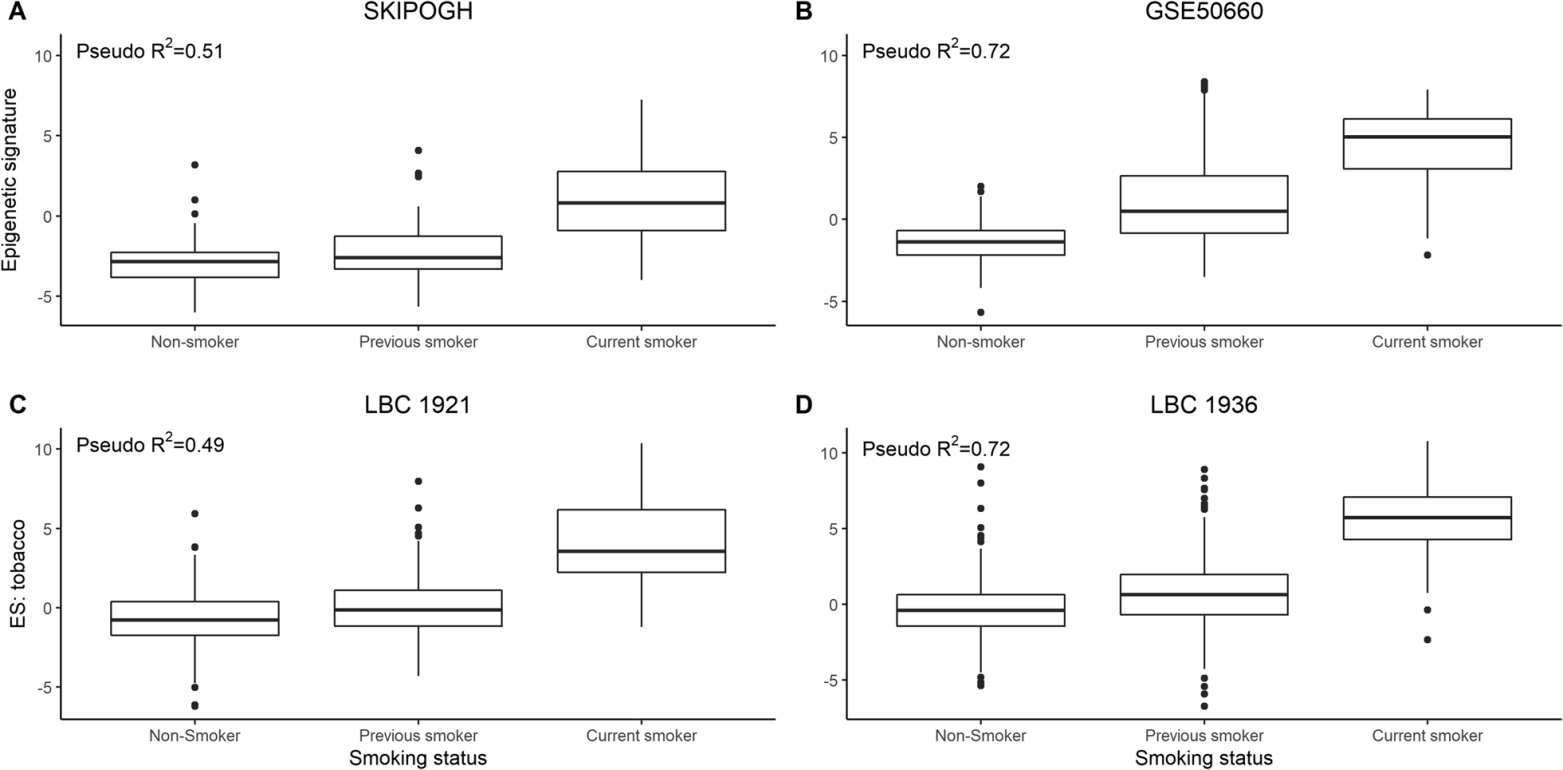
Distribution of SKIPOGH participants categorized by smoking status in function of their epigenetic signature**.** **A** Using data from the SKIPOGH cohort,** B** using data from the external validation cohort GSE50660,** C** using data from the LBC1921 cohort, and** D** the LBC1936 cohort. All reported pseudo-R2 values are from logistic regression models adjusting for age and sex

Among SKIPOGH participants, the epigenetic signature for tobacco consumption was positively associated with self-reported tobacco consumption for current or never smokers (McFadden’s pseudo-*R*^2^ = 0.51) (Fig. 1). When adjusting for age and sex, the epigenetic signature demonstrated a high capacity to distinguish between smokers and non-smokers in SKIPOGH (AUC = 0.92; 95% CI 0.90–0.95). In comparison, the EpiAlc signature had limited goodness-of-fit when distinguishing alcohol consumption non-drinkers to drinkers in the SKIPOGH (McFadden’s pseudo-*R*^2^ = 0.04), although this improved when restricting to heavy versus non-drinkers (McFadden’s pseudo-*R*^2^ = 0.24), and also in relation to standardized units consumed per week (SKIPOGH, adjusted *R*^2^ = 0.21).

### Validation

**Fig. 2:**
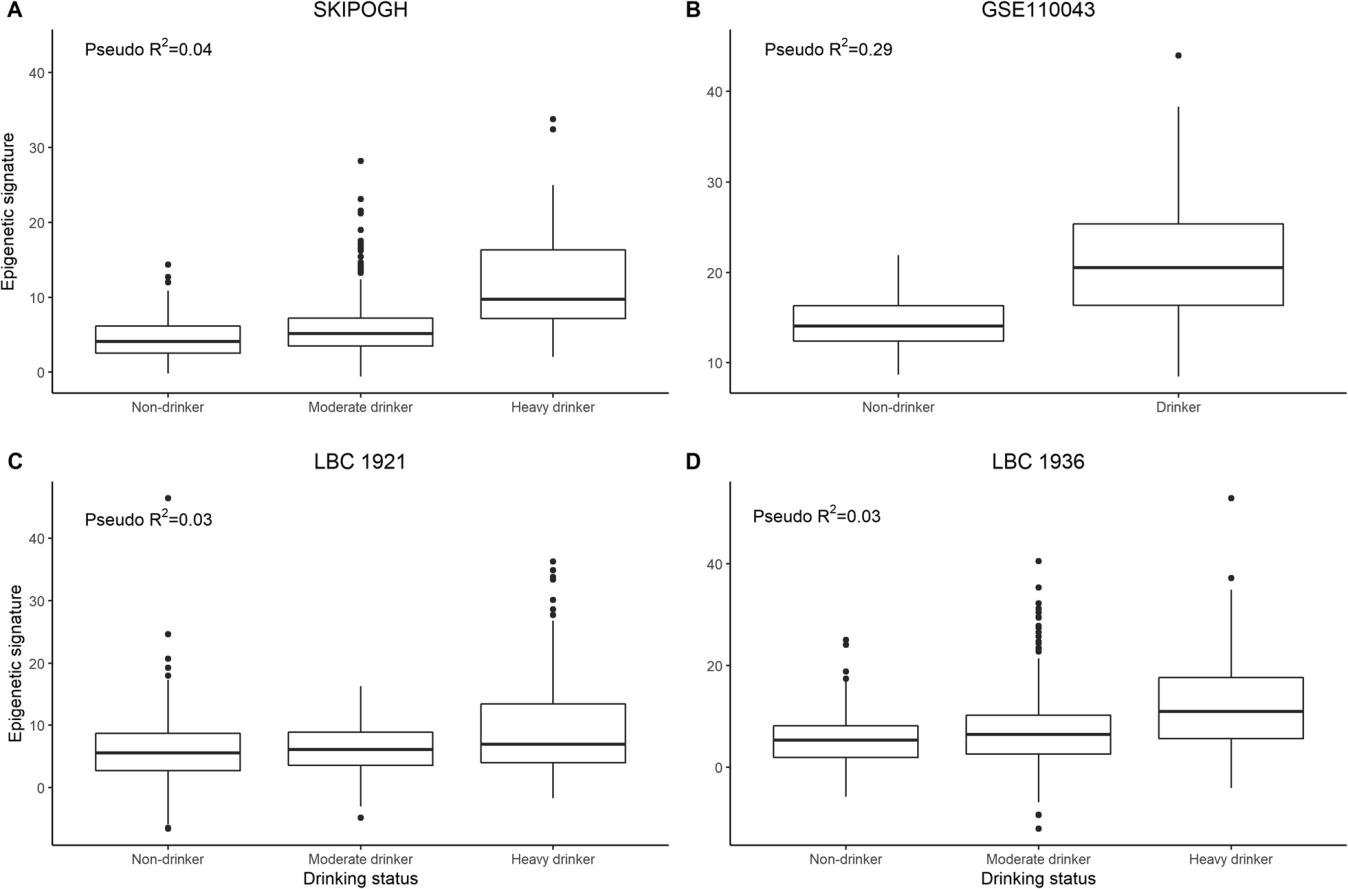
Distribution of SKIPOGH participants categorized by drinking status in function of their epigenetic signature. ** A** Using data from the SKIPOGH cohort,** B** using data from the external validation cohort GSE110043,** C** using data from the LBC1921 cohort, and** D** the LBC1936 cohort. All reported pseudo-R^2^ values are from logistic regression models adjusting for age and sex (except for the GSE110043 cohort, which only includes sex)

In comparison to the SKIPOGH cohort, the goodness-of-fit for EpiTob was even higher for the GSE50660 validation cohort (pseudo-*R*^2^ = 0.72), as well as the LBC1936 cohort (pseudo-*R*^2^ = 0.72). Goodness-of-fit for the LBC1921 cohort was like that observed in the SKIPOGH cohort (pseudo-*R*^2^ = 0.49) (Fig. 1). A high capacity for distinguishing between smokers and non-smokers was demonstrated for the LBC1936 cohort (AUC = 0.97; 95% CI 0.95–1.00). When comparing non-drinkers to drinkers in the LBC1921 and LBC1936 cohorts, the EpiAlc signature had limited goodness-of-fit (pseudo-*R*^2^ = 0.03 and 0.03, respectively) (Fig. 2), although this again improved when considering the standardized units consumed per week (LBC1921 *R*^2^ = 0.15; LBC1936 *R*^2^ = 0.21). The goodness-of-fit was considerably higher in the GSE110043 validation cohort when discriminating between heavy drinkers versus non-drinkers (pseudo-*R*^2^ = 0.29) (only adjusting for sex due to data availability). Finally, adjusting for age and sex, the epigenetic signature for alcohol consumption had a high capacity to discriminate between non-drinkers versus heavy drinkers (AUC = 0.74; 95% CI 0.67–0.81), which was slightly lower when discriminating between current drinking status (yes/no) (AUC = 0.64; 95% CI 0.60–0.68) in the SKIPOGH cohort.

### Association with cardiovascular disease

**Fig. 3:**
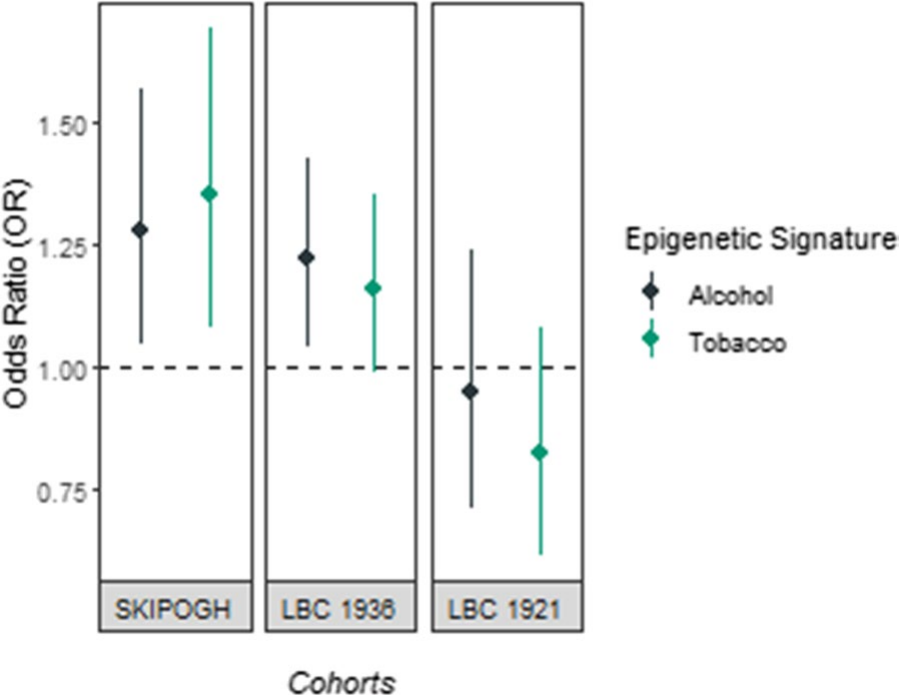
Odds ratios and 95% confidence intervals for the association between epigenetic signatures and CVD, stratified by cohort. Cohorts are organized according to the average age of the cohort. Average age of cohort participants for SKIPOGH = 50.9 years; LBC1936 = 69.7 years; LBC1921 = 79.3 years. Presented odds ratios correspond to the standardized signatures

Results from the logistic regression models of the association between EpiTob and EpiAlc with CVD are summarized in Fig. 3, stratified by cohort. When adjusting for age, sex, BMI, self-reported drinking status and education, a one standard deviation increase in the EpiTob signature was associated with a 31% higher odds of CVD in the SKIPOGH cohort (OR = 1.31, 95% CI 1.04–1.64), and a 16% increased odds in the LBC1936 cohort, albeit not significant (OR = 1.16, 95% CI 0.99–1.35). In contrast, a one standard deviation increase in the epigenetic signature for alcohol consumption was associated with a 28% increased odds of CVD in the SKIPOGH cohort (OR = 1.28, 95% CI 1.04–1.56), and a 22% increased odds in the LBC1936 cohort (OR = 1.22, 95% CI 1.04–1.43) (adjusting for self-reported smoking status) (Fig. 3). No statistically significant association was observed in the LBC1921 cohort for either alcohol or tobacco consumption (*p* value > 0.05), although higher levels of the ES for tobacco appeared to be associated with lower odds of reported CVD history (Fig. 3). In a sensitivity analysis restricting to individuals 70 years or older—to facilitate comparability between the SKIPOGH and LBC cohorts—the ES of tobacco consumption was no longer associated with CVD (OR = 1.07, 95% CI 0.89–1.27). Finally, inclusion of inverse probability weights did not modify the direction of results in the SKIPOGH cohort, but rather augmented the effect size for the ES of tobacco consumption (OR = 1.24, 95% CI 1.16–1.32).

### Risk of mortality

**Fig. 4:**
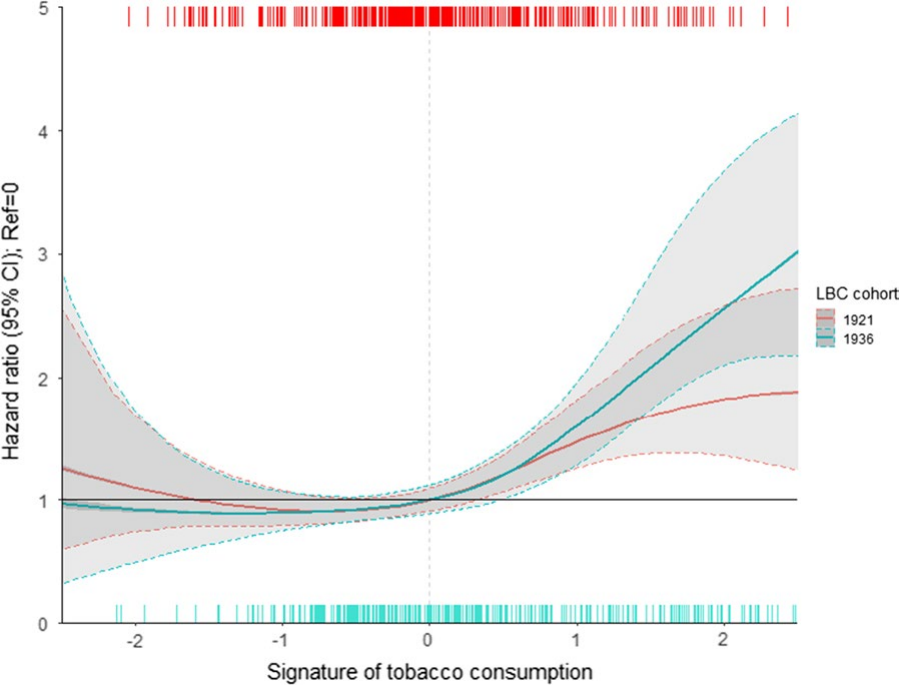
Risk of mortality according to level of epigenetic signature. Presented hazard ratios correspond to the standardized signatures. Dashed lines represent deaths from all-cause mortality at individual values of the epigenetic signature (so-called rug plot), whereby lines along the top of the figure correspond to deaths observed in the LBC1921 cohort, and along the bottom deaths observed in the LBC1936 cohort

An increase in the epigenetic signature for tobacco consumption was associated with an increase in all-cause mortality in Cox regression models for both the LBC1921 and LBC1936 cohorts (*centered at* 0) (Fig. 4). Following adjustment for smoking status, the epigenetic signature for alcohol consumption was not associated with all-cause mortality.

**Fig. 5:**
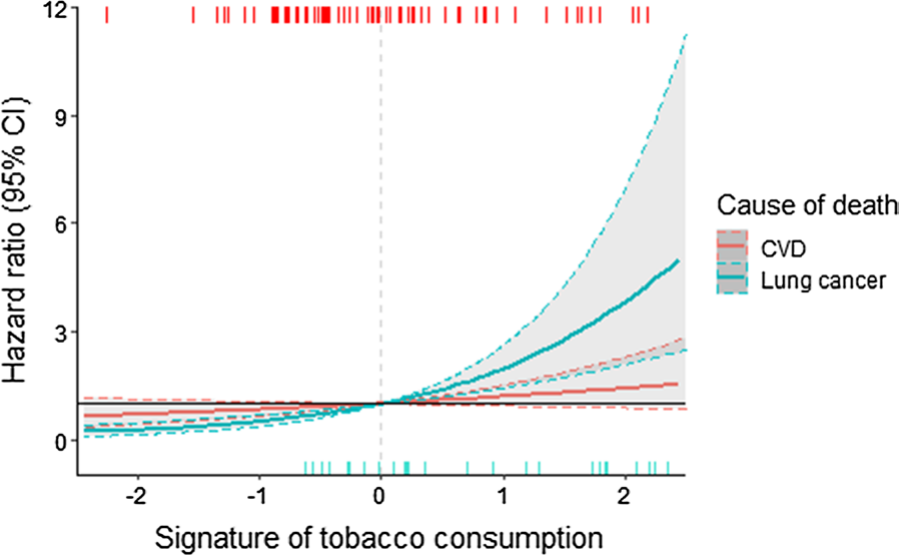
Cause-specific subhazard ratios for CVD and lung cancer-associated mortality. Each line corresponds to a separate model. Both models were adjusted for: age at entry, sex, BMI, years of education, and alcohol consumption. Presented hazard ratios correspond to the standardized signatures. Dashed lines represent cause-specific deaths at individual values of the epigenetic signature (so-called rug plot), whereby lines along the top of the figure correspond to CVD-related death, and along the bottom lung cancer-related death

A secondary analysis using the competing risk framework, increasing scores of the EpiTob signature were associated with an increasing mortality due to lung cancer in the LBC1936 cohort (Fig. 5). For example, a one standard deviation increase in the EpiTob signature was associated with a nearly two times higher risk of lung cancer-associated mortality (sHR = 1.95, 95%CI 1.45–2.64), but was non-significant for CVD (sHR = 1.18, 95% CI 0.93–1.51). A similar pattern was observed in the LBC1921 cohort, for which a one standard deviation increase in the ES of tobacco consumption was associated with a two-fold higher risk of lung-cancer associated mortality (sHR = 2.02, 95%CI 1.41–2.90). However, no association was observed between EpiTob or EpiAlc and CVD-associated mortality in the LBC1921 cohort (*p* > 0.05).

## Discussion

This study describes and validates two novel epigenetic signatures of lifestyle exposures. Moreover, the epigenetic signature for tobacco consumption was associated with an increased odds of self-reported CVD, as well as an increased risk of all-cause and lung cancer-related mortality in an independent cohort. Although the signature for alcohol consumption was also associated with an increased odds of reported CVD, it was not associated with all-cause or cause-specific mortality in the LBC cohorts.

The EpiTob and EpiAlc signatures generally required fewer CpGs and had a similar if not higher correlation with their associated self-reported phenotype (i.e., tobacco and alcohol consumption, respectively) in comparison with previously published epigenetic signatures. For example, Liu et al. proposed four epigenetic signatures for alcohol consumption (grams per day + 1) that were based on 5, 23, 78 and 144 CpGs [8]. Ignoring results based on the initial training dataset, the signature composed of 144 different CpGs explained the most variance (difference in reported R^2^ between the model with and without the epigenetic signature = 13.8) (data from the Atherosclerosis Risk in Communities study [19]), while the maximum variance explained by the five CpG-based signature was about 10% when using data from the LBC1936 cohort (*R*^2^ = 10.4). In comparison, the three CpG-based EpiAlc signature accounted for 13% of the explained variance when also using data from the LBC1936 cohort (adjusting for age, sex and BMI as done in the study by Liu et al. [8]). Directly comparing to the methylation scores of tobacco consumption estimated using the *EpiSmokEr* package [20], the EpiTob signature had a similarly high performance (*Additional file **1*). For example, using data from the LBC1936 cohort, the 187-CpG signature proposed by Elliott et al. explained 72% of the variance, while the 4-CpG score from Zhang et al. explained 77% [21, 22]. Notably, there was no difference in the predictive capacity of these signatures in comparison to the EpiTob signature (*p* > 0.05, adjusting for age and sex). There was similarly no difference between the predictive capacity (*p* value > 0.05) of the EpiTob signature—nor the signatures proposed by Elliott et al. or Zhang et al. —in comparison with the single-CpG approach (cg05575921)for the LBC1936 cohort [23]. Notably, included in the EpiTob signature is the same AHRR-associated CpG, cg05575921. Additional research is needed to determine whether the inclusion of additional CpGs beyond cg05575921 contributes to increased stability over time, lending to a more stable signature required for longitudinal assessments of exposure.

### Biological interpretation

The five CpGs for the signature of tobacco consumption were located on three genes (NDE1, CACNA1D, and AHRR), while the three CpGs composing the signature of alcohol consumption were located on two genes (SLC7A11, JDP2). The CpG sites included in both epigenetic signatures were initially identified through a literature search of published EWAS results that identified upwards of 3,000 CpGs (alcohol and tobacco consumption combined). In creating these epigenetic signatures, the models were maximized to ensure parsimony with the least amount of variables possible, while concurrently ensuring the greatest variance explanation as possible. Thus, the selected CpGs likely only capture a small portion of the whole, representing a few cogs in a greater physiologic machinery, and so it is unsurprising that the Gene Enrichment analysis identified neither significant terms nor necessarily relevant terms (see Additional file 1: Table). However, with respect to the annotated genes, the aryl hydrocarbon receptor repressor (AHRR) gene encodes a protein involved in the aryl hydrocarbon receptor (AhR) signaling cascade, which links environmental chemical stimuli (e.g., smoking) with adaptive responses [24]. In terms of the EpiAlc signature, the Solute Carrier Family 7 Member 11 (SLC7A11) gene is involved in the transport of cysteine and glutamate. A recent study by Lohoff et al. [25] used data from a population-base cohort, a case–control study, a postmortem mRNA analysis of human brain samples, and an mRNA analysis of liver tissues from mice, to provide evidence for the downregulation of SC7A11 in association with alcohol consumption; such downregulation has also been linked to increased oxidative stress. Moreover, Lohoff et al. [25] also identified CpGs annotated to the c-Jun-dimerization protein 2 (JPD2) gene in association with alcohol consumption.

### Utility of epigenetic signatures in epidemiological research

Cohort studies are often hindered by the type of data collected, unmeasured confounding, reliability of self-reported data and its associated biases (e.g., recall or response bias), as well as duration and intensity of exposure. Epigenetic signatures of lifestyle exposures, health conditions, and as predictors for disease risk and progression have the potential to assuage such issues in epidemiological research. Currently, objective biomarkers of exposure are available that measure self-reported exposure to tobacco consumption (e.g., cotinine levels with a half-life of 12–20 h) or alcohol consumption (e.g., ethyl-glucuronide [EtG] with a half-life of about 2–3 h) [26–29]. However, these biomarkers require additional tests, independent from one another, that can contribute to participant burden. Importantly for prospective cohort studies, participant burden can play a role in study attrition, which in turn may lend to a loss of power or even bias study results resulting in erroneous conclusions [30]. Moreover, measuring at a mechanistic level—or biological level—the impact of an exposure can aid in refining risk stratification in epidemiological studies, and in turn improve the identification of individuals most susceptible to developing disease.

### Utility of epigenetic signatures in clinical setting

Epigenetic signatures could become means to track intervention effectiveness of behavioral changes at an individual-level, which would be relevant in the clinical management of tobacco and alcohol use disorders, by providing a call for action and motivational tools. The EpiTob and EpiAlc signatures were created to maximize prediction and minimize the number of CpGs included; thus, although the choice to restrict to seven CpGs was arbitrary, the main goal was to drastically minimize the number of included CpGs. To this point, in terms of clinical utility, a parsimonious signature is advantageous as it can use less expensive techniques than chip arrays. Advancements in technology that are already underway (such as those by Oxford Nanopore technologies® and MassARRAY®) could facilitate measurement of only those CpG sites of interest, helping drive down costs and ensure accessibility. However, usage of these signatures to track the effectiveness of behavioral changes would be dependent on the plasticity of measured CpGs to changes in exposure. Although the plasticity of the epigenetic signatures could not directly be demonstrated in the present analysis, there are arguments for its existence. For example, in a longitudinal analysis Dugué et al. observed that changes in the reported alcohol intake were associated with changes in methylation levels for 513 CpG sites; within which two of the three CpGs (cg06690548 and cg00716257) encompassed within the EpiAlc signature were included [30]. In a separate analysis using data from the Framingham Heart Study, Liu et al. observed that methylation levels among heavy drinkers revert to that of non-drinkers by four years [8]. However, these results were based on samples taken at roughly four-year intervals, which makes it difficult to quantify a precise timeline of signature plasticity.

Similar to epigenetic changes induced by alcohol consumption, epigenetic changes following smoking cessation also appear to be relatively dynamic [32]. For example, using data from the Generation Scotland: Scottish Family Health Study—a large, population-based cohort—McCartney et al. [33] found that among light (so-called low-dose) smokers, only prolonged exposure to tobacco consumption induced epigenetic changes that could adequately characterize smoking status. Additionally, while it took less than a year for the methylation profile of low-dose ex-smokers to convert to that of a never smoker, it took up to nine years for high-dose ex-smokers [33]. Further demonstrating this plasticity, a recent study of adolescents observed that methylation levels of the cg05575921 CpG site (included in the EpiTob signature) remained stable over the course of two years among non-smokers, but diminished for smokers observed even within a 6-month period [34]. However, while the current evidence base is promising in terms of the clinical utility of EpiAlc and EpiTob to track progress of cessation efforts results, additional research is needed to prove the plasticity of these signatures and better understand the dose–response relationship. A final note regarding the utility of these—as well as other—epigenetic signatures in the clinical setting is the need to address ethical concerns surrounding the use of this sensitive information, most notably with regards to privacy and confidentiality [35]. While many countries have legislation in place that protects genetic information garnered from widely accessible genetic testing, such laws may need to be updated to address concerns unique to epigenetic information.

### Strengths and limitations

The epigenetic signatures included in the present manuscript were initially created using data from the SKIPOGH cohort. The SKIPOGH cohort is a population-based cohort including participant pairs or families with shared genetics. The genetic homogeneity and limited size of the SKIPOGH study population may be a potential limitation to the present study as it could have contributed to the lack of identification of relevant SNPs for inclusion in the final signatures, particularly given that genetic associations generally have very small effect sizes and hence would require a much larger sample size [36]. Similarly, the cohorts included in the present study are primarily composed of European populations. Recent evidence points towards an influence of ancestry on the epigenetic architecture that is largely driven by genetics, and has also evidenced differential methylation patterns according to ancestry [37], 38. As such, additional validation in a diverse population is necessary to ensure the generalizability to populations of non-European descent.

A strength of the present study is the validation of epigenetic signatures in independent samples with varied populations in terms of inclusion criteria, mean sample age, and background. To this effect, with respect to mean sample age, comparisons across the SKIPOGH and LBC cohorts may provide insights into the influence of bias on study outcomes. For example, while the epigenetic signatures for alcohol and tobacco consumption were associated with self-reported CVD in the SKIPOGH cohort, the strength of these associations appeared weaker in the LBC1936 cohort, and was non-existent in the LBC1921 cohort. The diminishing strength of association across the three cohorts may be influenced by the higher average age, and as such a result of survivor bias. This is similar to the observed fact that epigenetic age increases at a slower rate than chronological age, especially among older individuals, which is likely a consequence of survival bias [39]. Moreover, both LBC cohorts have proportionally fewer smokers at baseline, which is also likely influenced by the older age of cohort participants. Another potential limitation of the present study that may have affected comparability across studies was that normalization techniques of the DNA methylation data were not standardized across the included studies and samples, nor did all included samples use the same methylation profiling array. For example, DNA methylation data for the Lothian Birth Cohorts was obtained using the Infinium® HumanMethylation450 BeadChip assay [13], while the majority of the SKIPOGH participant methylation data was obtained using the Infinium® Methylation EPIC BeadChip. However, regardless of the array used, replication results suggest that the signatures are robust to the potential added noise associated with varying normalization techniques. Finally, self-reported levels of alcohol and cigarette consumption were used to assess the relationship between self-reported tobacco or alcohol consumption and the respective epigenetic signature. No validation study for either the SKIPOGH or LBC cohorts has confirmed the reliability of self-reported alcohol or tobacco consumption. Therefore, as self-reported data are subject to self-report or recall bias, e.g., smokers may underreport their tobacco intake levels, effect estimates may be over- or underestimated. This could have contributed to a less than optimal choice of CpGs selected for inclusion in the signature. However, the association of the included epigenetic signatures with long-term health outcomes such as self-reported CVD or mortality suggests the validity of the included CpGs as biomarkers for the long-term effects, although they are based on self-reported exposures.

## Conclusion

This study shows the potential of two novel simple epigenetic signatures to measure self-reported exposure status and their clinical relevance given the association with long-term health outcomes. Measurement of lifestyle exposures via epigenetic signatures may assuage measurement issues and biases experienced in cohort studies and, perhaps more importantly, could aid in risk stratification for informing targeted interventions. However, future research is needed to clarify the external validity of these novel epigenetic signatures and also ensure the generalizability of these signatures to populations of non-European descent.

## Supplementary Information

**Additional file 1**. **Table 1** presents a list of the ICD-10 codes used to identify cause-specific mortality outcomes.** Table 2** presents the SKIPOGH population characteristics stratified according to epigenetic data availability.** Table 3** provides AUC values for the EpiTob and EpiAlc signatures in comparison with previously published signatures estimated using data from the LBC1921 and LBC1936 cohorts.

## Data Availability

Data for the SKIPOGH cohort is available upon formal request submitted to the SKIPOGH steering committee (https://www.maelstrom-research.org/study/skipogh). Data for both LBC studies are available upon formal request, additional information can be found on the website: https://www.ed.ac.uk/lothian-birth-cohorts/data-access-collaboration. All epigenetic signatures are available upon request for non-commercial use by contacting genknowme at labo@genknowme.com.
